# Topological Ring Currents in Open-Shell Homologues of Clar’s Goblet and Triangulene

**DOI:** 10.1021/acs.jpca.5c06508

**Published:** 2025-12-08

**Authors:** Timothy K. Dickens, Roger B. Mallion

**Affiliations:** Peterhouse, Cambridge CB2 1RD, England, United Kingdom

## Abstract

The Hückel–London–Pople–McWeeny (HLPM) formalism for calculating “topological” ring currents was recently extended to make it applicable to open-shell π-electron-conjugated systems. This modification, which incorporates the device of “Configurational State Averaging” (also known as “Electron Smearing” and “Fractional Occupation”), was then used to calculate the topological ring currents associated with the 11-ring benzenoid diradical that has become known as Clar’s goblet. The present paper investigates the quantitative trends among the topological ring currents in some other classic diradicals, and other multiple radicals, that are now accessible via the HLPM approach, namely, certain homologues of Clar’s goblet and triangulene and several of their oxidation states, and compares their patterns and sizes with pictorial current maps generated by more sophisticated pseudo-π and *ab initio* calculations.

## Graphical abstract

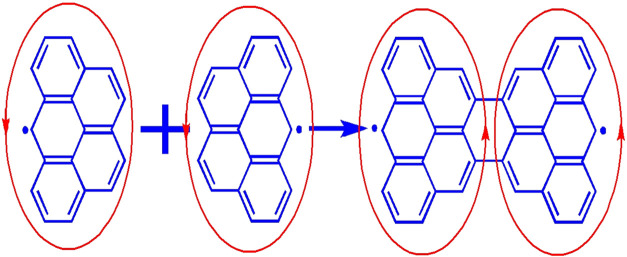

## Introduction

The Hückel–London–Pople–McWeeny (HLPM)^1^ approach for calculating “topological” ring currents and bond currents^1^ in π-electron-conjugated systems was recently extended^2^ to make it applicable to open-shell conjugated systems. The expressions presented in McWeeny’s original formulation^3^ of the Hückel–London theory^4^ for studying the π-electron magnetic properties of conjugated structures are valid only for closed-shell systems. This shortcoming was removed in ref 2 by judiciously applying the device of “Configurational State Averaging” (CSA);^2,5^ this technique (under the more intuitive label of “Fractional Occupation”) had earlier been applied in the context of alternative formulations^5,6^ of the London approach.^4^ The present paper will investigate the quantitative tends among the topological ring currents in some other classic radicals, namely, certain homologues of Clar’s goblet and triangulene and several of their oxidation states, and will compare their patterns and sizes with pictorial current maps generated by more sophisticated pseudo-π and *ab initio* calculations.^7,8^ For convenient reference, the connectivity of the carbon atoms in the two principal structures studied in this paper is displayed in the molecular graphs^1^ of Clar’s goblet and triangulene that are presented in Figure 1; the scheme for labeling their symmetrically distinct rings is also shown.

**Figure 1:**
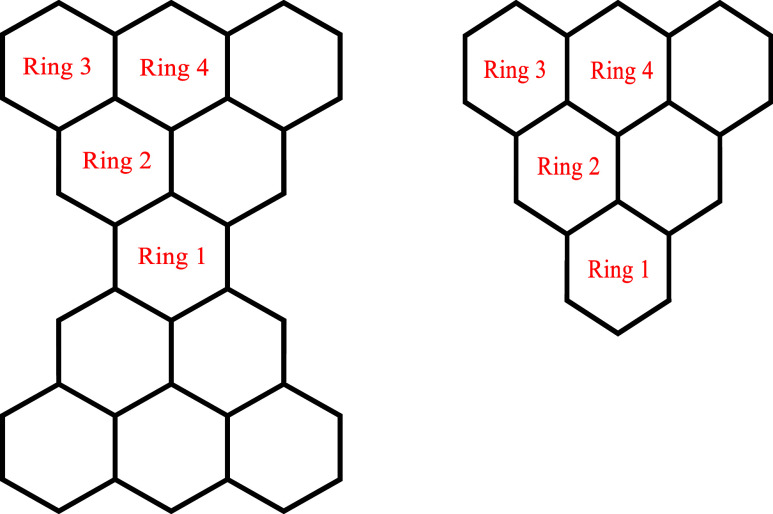
Molecular graphs, displaying carbon–carbon connectivity, of Clar’s goblet (left-hand side) and triangulene (right-hand side), together with the numbering schemes used to label the four symmetrically distinct rings that each of these structures possess.

It may be noted that within the last five years, there have been some exciting developments in the synthesis of these iconic conjugated systems.^9–11^

## Methods of Calculation

The topological ring currents reported in this work were all calculated by use of the HLPM formalism, as described and illustrated step-by-step in refs 1 and 12, but with the definition of imaginary bond–bond polarizabilities detailed on p 306 of ref 1 (eqs 12 and 13) replaced by eq 12 on p 10184 of ref 2. The device of “Configuration State Averaging” is invoked if, in the course of applying the *Aufbau* process, an open-shell situation arises whereby *p* degenerate energy levels are obliged to accommodate *q* π-electrons; the latter may then be “smeared” or “averaged” so that each of the *p* orbitals takes *q*/*p* electrons. The common occupation number (ν_*i*_) for each of these degenerate orbitals is thus *q*/*p*. The ratio *q*/*p* may, of course, be fractional, hence leading to the more formal and intuitive name of this maneuver (“Fractional Occupation”).

In addition to requiring knowledge of the carbon–carbon connectivity of the conjugated hydrocarbon under study (which is embodied in its molecular graph;^1^ see, for example, Figure 1), application of the HLPM approach also needs (a) actual or assumed values for the areas of the individual rings of the system and (b) an assumption that the structure in question is (geometrically) planar. These requirements are expected to be especially realistic in the present work because all of the structures considered here (homologues of Clar’s goblet and triangulene (Figure 1)) comprise tessellations of regular hexagons of carbon atoms that may be extended arbitrarily in the plane, *ad infinitum*. Furthermore, although Clar’s goblet and its homologues contain peripheral hydrogen atoms that, on Martin’s terminology,^13^ are called ‘H α 3 protons’, Coulson and Haigh’s studies on molecular overcrowding^14^ in phenanthrene, triphenylene, and chrysene showed that such protons do not cause sufficient steric hindrance to require deviation from the planarity of the structure as a whole. In addition, triangulene possesses no sterically interfering peripheral hydrogen atoms whatsoever. Hence, the HLPM assumption that the conjugated system under study is geometrically planar would appear to be well justified so far as Clar’s goblet and triangulene, and their respective homologues, are concerned.

The more sophisticated current maps with which the HLPM topological ring currents will be compared will be computed by use of the pseudo-π^7^ and *ab initio* ^8^ procedures described in refs 7 and 8. Unlike the HLPM approach, the ipso-centric method (which can be calculated, with varying degrees of approximation, from pseudo-π^7^ to *ab initio* ^8^) does not immediately provide simple bond current values that obey Kirchhoff’s rules or a single value for the current flowing in each ring. However, the ipso-centric approach does give a nuanced qualitative picture showing how the ring currents are actually flowing around discrete atoms.

## Results and Discussion

### Homologues of Clar’s Goblet

Figure 2 shows a scheme to indicate which homologues are being considered and the conventions adopted for labeling the constituent rings of each.

**Figure 2:**
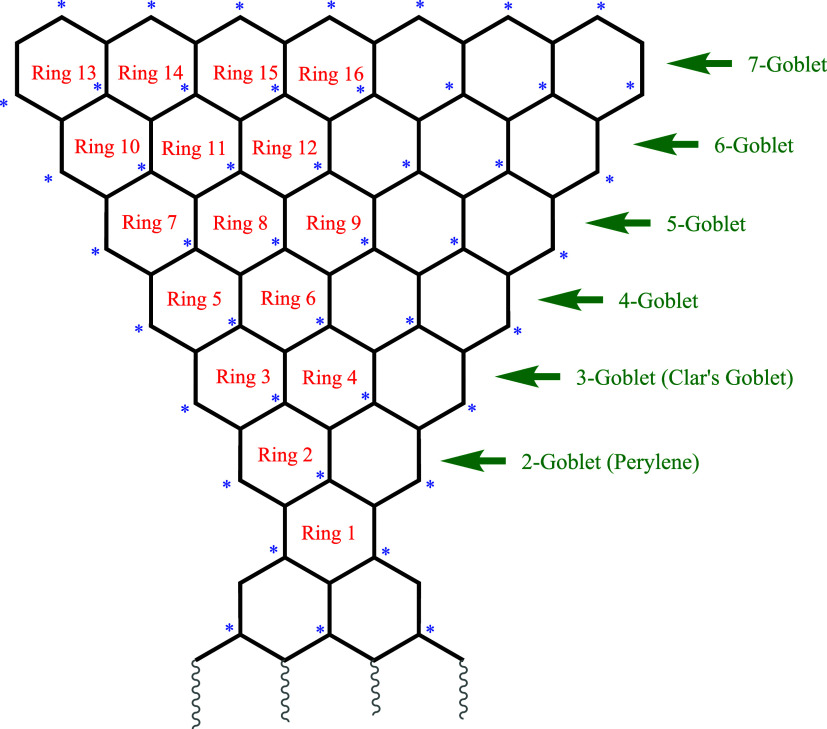
Schematic representation, and ring labelings, for several homologues (2-goblet to 7-goblet) of Clar’s goblet, all of which are alternant hydrocarbons with an even number of carbon atoms. On this convention, “2-goblet” is the (closed-shell) stable benzenoid hydrocarbon perylene and “3-goblet” is the (open-shell) diradical that is Clar’s goblet itself.

Table 1 presents the topological ring currents in the various rings of homologues 2-goblet (perylene), up to and including 7-goblet, of Clar’s goblet (3-goblet). Those cells highlighted in yellow indicate internal rings, that is, they have no external edge.

**Table 1:**
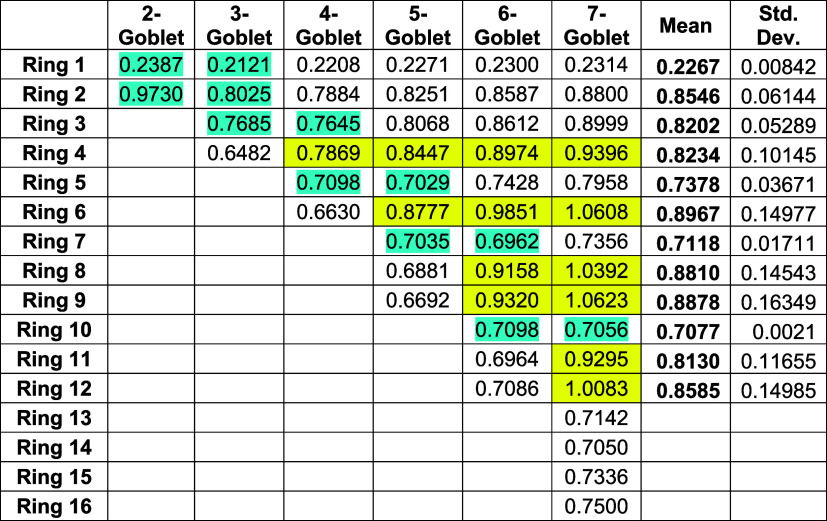
Topological Ring Currents (Expressed As a Ratio to the Benzene Value) in Neutral 2-Goblet to 7-Goblet

We note the following from Table 1:

(a) The most immediately striking aspect of Table 1 is that the central ring (Ring 1) in all of the goblets studied bears a significantly smaller ring current than any other ring. It is (approximately) only 20% of the benzene value. Such an observation concerning the topological ring current in the central ring of perylene has been made several times previously^15,16^ and the ring has been said to be an “empty ring” ^17,18^ containing two “fixed” single bonds^19,20^ when any Kekulé structure is depicted for the structure. This idea featured in Clar’s invocation^21,22^ of “bond fixation” in perylene in order to account for the low ring currents in the central ring that seemed to be indicated from experimental ^1^H NMR chemical shifts.^21^ Nevertheless, although the idea that “bond fixation” in the central ring explains a low ring current in that ring is intuitively appealing, the situation is not as simple as it seems.^2^ It should be noted that perylene is a closed-shell system for which Kekulé structures can be drawn (in all of which, as noted, two peripheral bonds are indeed “fixed” as single, in the central ring). However, *Clar’s goblet and its larger homologues are all radicals*, and so *there is no opportunity to draw a Kekulé structure for them*. Hence, the size of the ring currents in the central rings (which are equally as small as for the corresponding ring current in the central ring of closed-shell perylene)^15,16^ cannot be attributed to bond fixation in the central rings for the very reason that no such bond fixation can be demonstrated because *no Kekulé structures can in practice be realized for these structures*.
(b) One striking observation is that as the size of the goblet is increased, the ring current in any given position generally increases (highlighted in pale cyan color in Table 1).
(c) The standard deviation in the ring current for any given ring remains small as the size of the goblet is varied, though this is larger for those rings that are internal (that is, those that have no external edge), indicated by the yellow areas in Table 1.
(d) The largest observed ring currents in any given goblet are generally, though not universally, in those rings that do not have an external edge, as can be seen from the yellow area in Table 1.
(e) It has been noted in Figure 2 that 2-goblet is actually the closed-shell alternant hydrocarbon commonly known as perylene. The computations reported here (effected by double-precision calculation) predict the topological ring currents in perylene (to 3 significant figures) to be 0.239 for the central ring and 0.973 for each of the four outer rings. This is consistent with what, after several recalculations over the years,^23–26^ was eventually the settled view of the intensity of these topological currents (to the same accuracy) in the early literature.^23–26^

Table 2 displays the topological ring currents in rings 1−4 of neutral Clar’s goblet and in 12 of its oxidation states (cations with charges +1 to +6 and anions with charges −1 to −6).

**Table 2:**
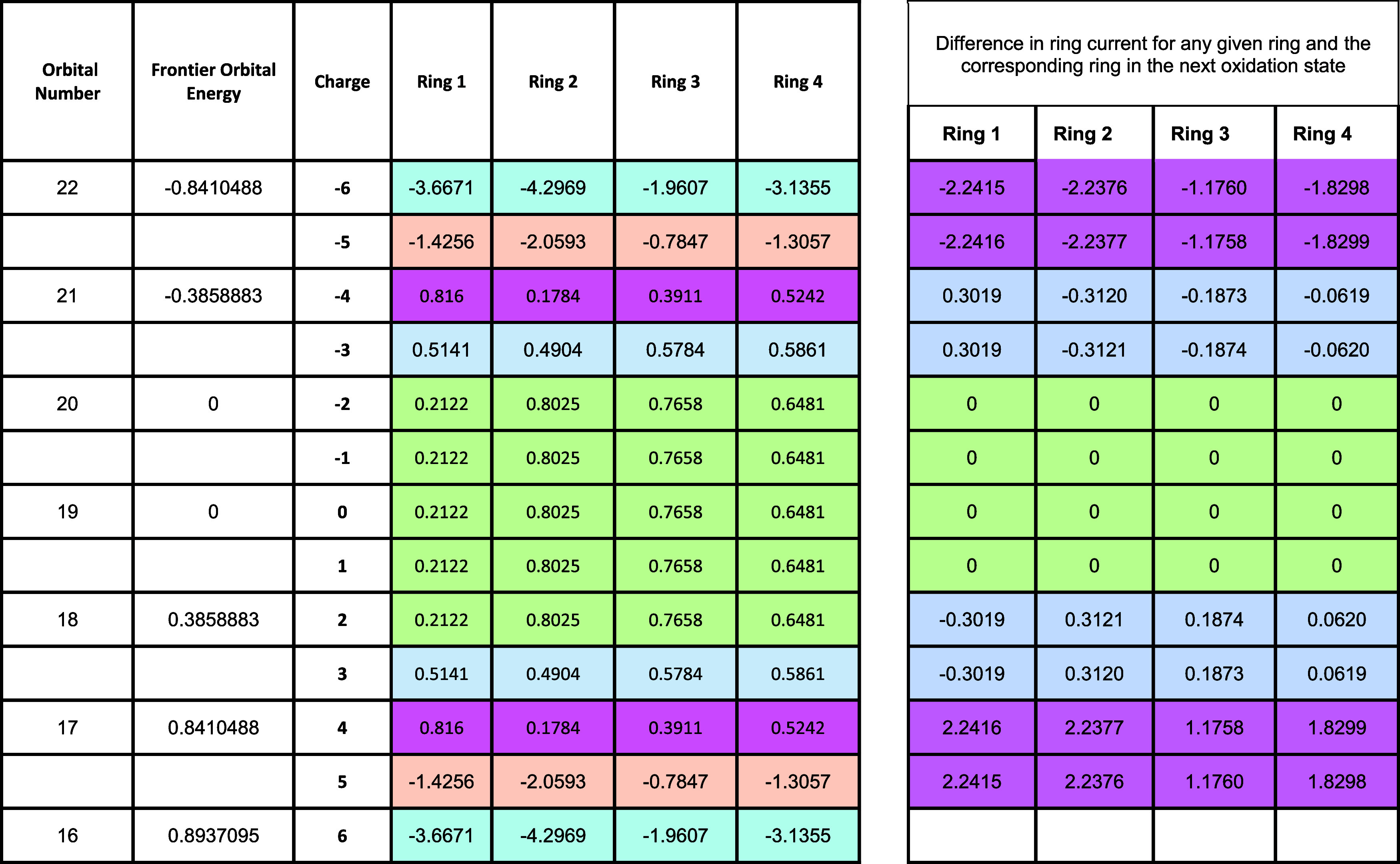
Topological Ring Currents in Rings 1−4 of Neutral Clar’s Goblet and 12 of Its Oxidation States (Cations with Charges +1 to +6 and Anions with Charges −1 to −6)

It might be helpful to the reader to recall that neutral 3-goblet has 38 π-electrons; when the *Aufbau* process and Hund’s rules are applied, 36 of these are accommodated by their completely occupying energy levels 1–18, and the remaining two π-electrons have to be assigned to the two degenerate nonbonding orbitals, 19 and 20, one in each.

The last four columns of the subtable on the right-hand side of Table 2 record the difference in the ring current between a given ring at a given charge and the ring current value at the next charged state. The ring currents change, but by the same amount, where the background color (magenta, blue/gray, or green in the right-hand subtable of Table 2) is the same.

The following observations may be made concerning Table 2:

(a) First, there is the remarkable finding that the topological ring currents in the following four oxidation states of the 3-goblet—the monoanion, the dianion, the monocation, and the dication—*are all identical with the respective ring currents calculated for the neutral species*. This is illustrated by examining the green-shaded area in the left-hand subtable of Table 2. This observation is reminiscent of a similar, but more limited, phenomenon that we observed some years ago in the context of certain *nonalternant* neutral and dianionic *altans*,^27,28^ to which we shall return later in the discussion.
(b) It may also be noted that the magnitude of the difference in the ring current between the −3 and −4 oxidation states and of that between the +3 and +4 oxidation states is likewise identical (as can be seen in the rows highlighted in magenta and in blue/gray in the subtable on the left-hand side of Table 2).
(c) There is a similar pattern (highlighted in the rows colored in pale cyan and in peach in the left-hand subtable of Table 2) regarding the −5 and −6 oxidation states and the +5 and +6 oxidation states.

Two observations about ring current variation with electron count can be made from the remarks (a)–(c) above. The first relates to the linearity of ring current contributions with partial filling of the orbital(s) at a fixed orbital energy (eigenvalue), and the second concerns the invariance of current with occupation of the nonbonding (zero eigenvalue) orbitals (Figure 3).

**Figure 3:**
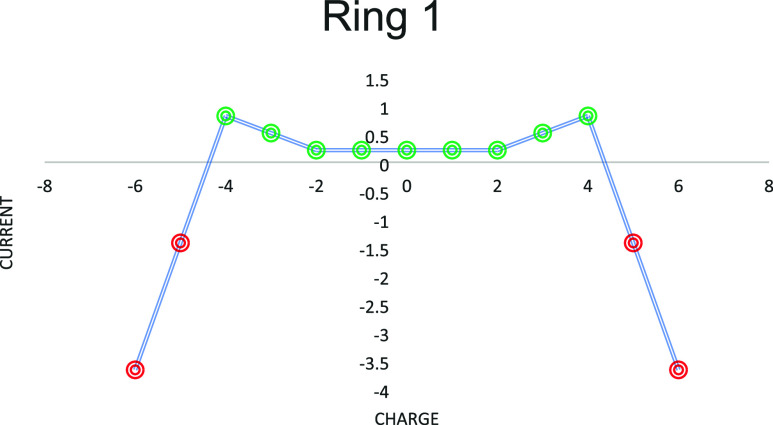
Ziggurat diagram^35^ showing the current in the central ring for various charged species of Clar's goblet. It should be noted (1) that the charts are all symmetric because we are dealing with bipartite graphs; (2) how the currents are all diamagnetic around the neutral and smaller charged species; and (3) as the charge is increased, the ring currents become heavily paramagnetic. Diamagnetic points are colored green, and paramagnetic points are colored red.

The first property follows directly from using the device of “Configuration State Averaging” (CSA),^2,5,6^ as discussed earlier in the Methods of Calculation section. This is because the imaginary bond–bond polarizability (defined in eq 12 of ref 2) is linear in fractional occupation; this applies to all Hückel systems.

The second is a specific feature of the CSA approach when it is applied to bipartite (alternant) systems such as benzenoids, and here, of course, the benzenoids that we explicitly consider are homologues of Clar’s goblet and triangulenes (both illustrated in Figure 1). It is well known that unweighted bipartite molecular graphs obey the Coulson–Rushbrooke pairing theorem.^29,30^ In the sum-over-states formula for the imaginary bond–bond polarizability (eq 12 of ref 2), an electron in a shell with a given eigenvalue and an electron in the shell with minus that eigenvalue thus make equal and opposite contributions to current. Consequently, linearity then implies that occupation of a nonbonding shell in an alternant hydrocarbon has no effect on the current map, when the latter is calculated within the CSA approach.^2,5,6^

A formal proof of this invariance, based on the Aihara formulation of the Hückel–London^4^ method (see, for example, refs 31 and 32), is due to Myrvold, Fowler, and Clarke in ref 6. They first prove a theorem concerning the circuit resonance energy of a cycle (as defined by Aihara^31,32^ ) and then draw attention to three corollaries of it.

**Corollary 1** asserts that, in the CSA model, paired shells of a bipartite graph that contain the same number of electrons make self-canceling contributions to the current and hence make no net contribution to the Hückel–London^4^ current map.

**Corollary 2** shows that, on the CSA assumptions, all electrons in a nonbonding shell of a bipartite graph (alternant hydrocarbon) likewise make no net contribution to the Hückel–London^4^ current map.

**Corollary 3** emphasizes that, on the CSA formalism, the Hückel–London^4^ current maps are identical for the *q* ^+^ cation and the *q* ^–^ anion of a π-system with a bipartite molecular graph.

As remarked earlier, in Table 2, several different colors have been used to draw readers’ attention to how calculated ring currents for the various charged species are paired, according to the Coulson–Rushbrooke theorem.^29,30^ (These colors are pale cyan, peach, magenta, blue-gray, and green.) In the subtable on the left of Table 2 it can be seen from an examination of the green area that placing electrons into the nonbonding orbitals makes no contribution to the current. This effectively exemplifies Corollary 2, above, of Myrvold et al.^6^

Corollary 3 is likewise illustrated in the subtable on the left of Table 2 by considering the hexaanion and hexacation (the rows in pale cyan), the pentaanion and the pentacation (the peach-colored rows), the tetraanion and the tetracation (the magenta rows), and the trianion and trication (the blue/gray rows). The green area (concerning the neutral species, the monoanion, the monocation, the dianion, and the dication) also serves to illustrate Corollary 3.

The subtable on the right of Table 2 reflects the difference in the calculated ring current between a given orbital and the calculated ring current in the next orbital. The pattern emerges that the differences in the ring current in a shell with one electron, or two, are identical, and furthermore, this difference is replicated but with the opposite sign, which arises for orbitals paired by the Coulson–Rushbrooke theorem.

(It should be noted that the CSA formulation neglects state-specific effects on current, such as those arising from Jahn–Teller splittings, which can in practice be substantial.^33,34^ )

Regarding Corollary 1, the contribution per electron from an occupied +λ bonding eigenspace is equal and opposite to the contribution per electron of an unoccupied λ antibonding eigenspace. Hence, for a molecule with a bipartite molecular graph *G*, the currents are identical for the *q* ^+^ cation and the *q* ^–^ anion. In this sense, bond currents and ring currents all have symmetrical “ziggurat” profiles^35^ against electron count, where each eigenspace is a ramp (of positive, zero, or negative slope), and, in particular, the nonbonding shell gives zero current per electron in a flat top to the ziggurat.

Tables analogous to Table 2 were prepared for 2-goblet (a.k.a. [closed-shell] perylene), 4-goblet, and 5-goblet (please see Figure 2 for illustrations of these species; but these tables are not reproduced here). Suffice it to say that, for *n*-goblet, the following two quantities increase as *n* increases: the number (μ) of nonbonding orbitals and the number (*s*) of oxidation states having identical ring currents. At least for those initial members of the series (shown in Table 3) *that are open-shell* (that is, for *n* > 2), these quantities are connected by the relation *s* = 2μ + 1. Prediction of the number (μ) of nonbonding orbitals is given by μ = 2*n* – 4. By combining them, these relations can also be expressed as *s* = 4*n* – 7. The observation that these relations do not hold in 2-goblet is intuitively consistent with the fact that this species is a “well-behaved”, closed-shell, benzenoid hydrocarbon, with no nonbonding orbitals, and so it has no diradical status, and (unlike the rest of the series) it, thereby, possesses Kekulé structures.

**Table 3:** Numbers (μ) of Nonbonding Orbitals and Numbers (s) of Oxidation States Having Identical Ring Currents in *n*-Goblet (*n* > 1)

*n*-goblet	no. (μ) of nonbonding orbitals	no. (*s*) of oxidation states having identical ring currents
2-goblet	0	0
3-goblet	2	5
4-goblet	4	9
5-goblet	6	13
6-goblet	8	17
7-goblet	10	21

In order to test the suitability of the rudimentary Hückel–London (HLPM) approach,^1,4^ the 2-goblet and 3-goblet structures were also investigated using an *ab initio/*ipso-centric^8^ and a pseudo-π approach.^7^ This yielded the ring current diagrams for neutral 2-goblet (perylene) shown in Figure 4.

**Figure 4:**
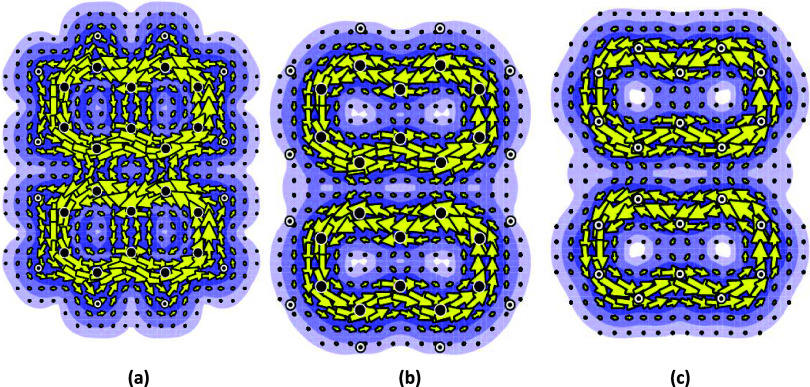
Current maps from ipso-centric^8^ and pseudo-π^7^ approaches for neutral 2-goblet (perylene). (a) Ring current map derived from both σ- and π-orbitals using the *ab initio* method;^8^ (b) ring current map derived from just the π-orbitals using the *ab initio* method;^8^ and (c) ring current map calculated using the pseudo-π method.^7^

It should be noted that there is little ring current associated with the central ring. The ring currents behave to a first approximation as if the structure were two separate and isolated naphthalene units with diamagnetic ring currents. This is all in accordance with Clar’s view,^21^ and that of others,^15–20^ some from many years ago.^15,16^ Calculating ring currents associated with just the π orbitals yields the current map depicted in Figure 4b.

It can clearly be seen that what little ring current there is in the central ring is almost exclusively associated with the σ-orbitals. The calculation was repeated using a pseudo*-*π approach. This yielded an almost identical picture (Figure 4c).

3-Goblet was explored in a similar fashion. The dication, neutral species, and dianion were examined and yielded the diagrams illustrated in Figure 5.

**Figure 5:**
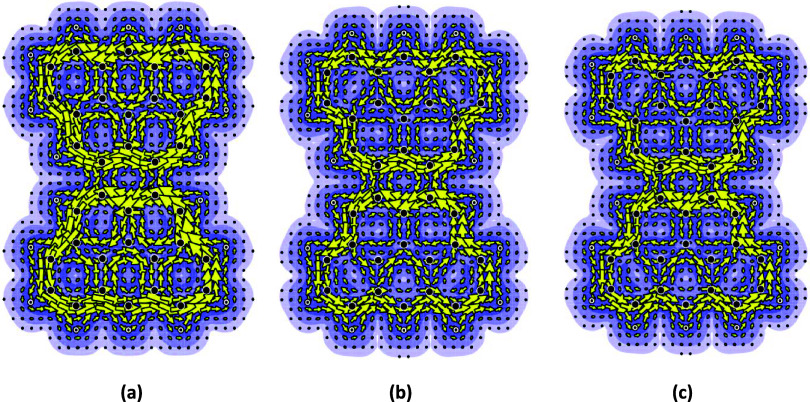
Current maps from an *ab initio/*ipso-centric approach^8^ for 3-goblet. (a) Ring current map of the dication with σ- and π-contributions; (b) ring current map of the neutral molecule with σ- and π-contributions; and (c) ring current map of the dianion with σ- and π-contributions.

In all three species based on 3-goblet, it should be noted that the ring current in the central ring is small, just as with the treatment of the 2-goblet structure. When only the π-electron contributions are considered, it can be seen from Figure 6 that there is effectively no contribution to the ring current in the central ring.

**Figure 6:**
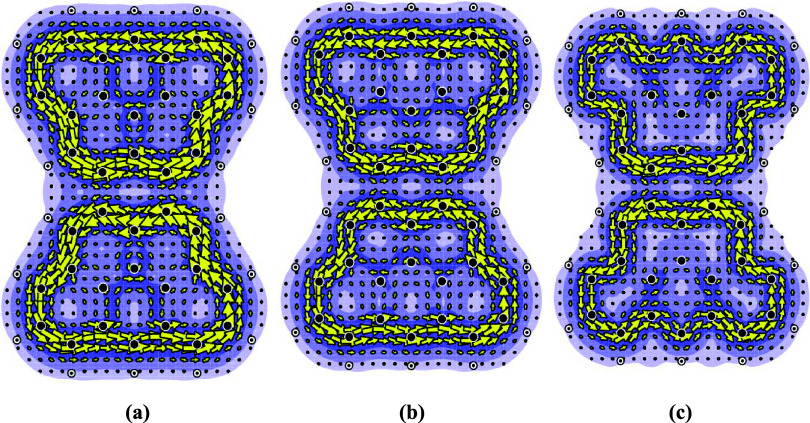
Current maps from an *ab initio/*ipso-centric approach^8^ for 3-goblet, with π-contributions only. (a) Ring current map of the dication with π-electron contributions only; (b) ring current map of the neutral molecule with π-electron contributions only; and (c) ring current map of the dianion with π-electron contributions only.

It should be noted that in all cases, the currents are similar in magnitude and indicate flow in the same counterclockwise (diamagnetic) direction. Pseudo-π calculations yielded very similar diagrams (and they are therefore not reproduced here). It should be borne in mind that the energies of the frontier orbitals (and, indeed, the family of energies of all of the other orbitals) in all three species—the neutral, dianionic, and dicationic 3-goblets—are identical, on the Hückel model; the differences in the ipso-centric current maps associated with each of them are due to electron correlation effects (that is, electron–electron interactions) that a Hückel approach simply does not evaluate.

### Homologues of Triangulene and Truncated Triangulene

Figure 7 shows a scheme to indicate which homologues of triangulene are being considered here and the conventions that are adopted for labeling the constituent rings of each. These structures also intrigued Clar,^21^ who claimed on p 112 of ref 21 that “··· hydrocarbons built up of hexagons in a triangular shape must be radicals··· ”. Clar also noted that all such species are alternant hydrocarbons. Because of the Coulson–Rushbrooke theorem,^29,30^ the last statement means that the triangulenes with an odd number of carbon atoms must have at least one nonbonding orbital and, in general, such species must possess an *odd number* of such nonbonding orbitals.^25,29,30^ Examples are 2-triangulene (a monoradical with 1 nonbonding orbital) and 4-triangulene (a triradical with 3 nonbonding orbitals). Alternant hydrocarbons with an even number of carbon atoms, if they do have any nonbonding orbitals, must have an even number of them.^29,30^ Examples are 3-triangulene (a diradical with 2 nonbonding orbitals) and 5-triangulene (a 4-fold radical with 4 nonbonding orbitals). It has been noted earlier in this article that *all* homologues of Clar’s goblets are alternant hydrocarbons with an *even* number of carbon atoms. However, not all *n*-triangulenes have an even number of carbon atoms; only every *other* one does.

(i) 2-Triangulene has an *odd* number of carbon atoms (with 13 carbon centers).
(ii) 3-Triangulene is an *even* alternant benzenoid (with 22 carbon centers).
(iii) 4-Triangulene is an *odd* alternant benzenoid (with 33 carbon centers).
(iv) 5-Triangulene is an *even* alternant benzenoid (with 46 carbon centers).

**Figure 7:**
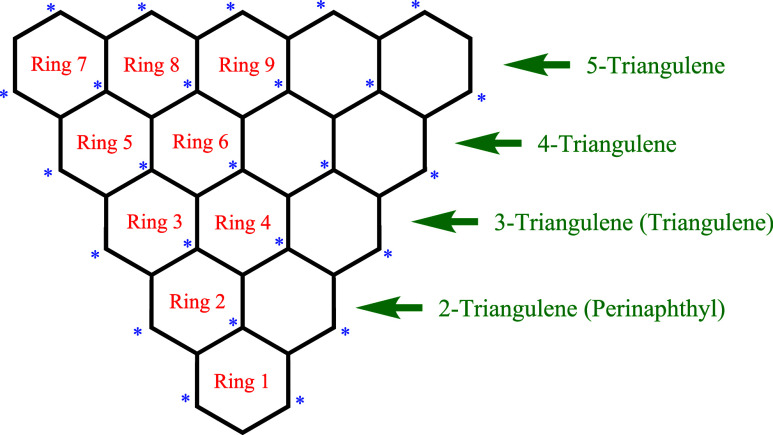
Schematic representation, and ring labelings, for several homologues of triangulene (2-triangulene to 5-triangulene). On this convention, “3-triangulene” is the (open-shell) diradical that is elsewhere^21^ known simply as triangulene; the first member of the series (with *n* = 2), the three-ring monoradical 2-triangulene, is also known^21^ as phenalyl or perinaphthyl.^21^

From the above data, proof by induction reveals that the number of carbon centers (*c*) in *n*-triangulene (*n* > 1) is given by *c* = *n* ^2^ + 4*n* + 1. Note from the preceding expression that when *n* is even, *c* is odd and that when *n* is odd, *c* is even. It may further be remarked that benzene can formally be considered as “1-triangulene” (with six carbon centers, as *per* the above formula when *n* = 1).

Clar likewise studied what, in the nomenclature introduced in Figure 7, are called 4-triangulene and 5-triangulene; these are Clar’s structure (XXXI) on his labeling scheme^21^ (a triradical) and (XXXII) (a 4-fold radical), respectively, discussed on p 112 of ref 21. The first member of the triangulene series (with *n* = 2) is a monoradical consisting of just three rings that would be called 2-triangulene on the scheme adopted in Figure 7 and which is more widely known as phenalyl or perinaphthyl.^21^

Table 4 presents the HLPM^1,2^ topological ring currents in the various rings of homologues 2-triangulene (otherwise known as^21^ phenalyl or perinaphthyl) up to and including 5-triangulene. The cell highlighted in yellow indicates one internal ring; that is, this ring has no external edge. Note that this internal ring, Ring 4, of 5-triangulene, bears the largest ring current in the structure. It may be observed from Table 4 that all ring current intensities in all four triangulene homologues studied here are less than the benzene value (of 1.0000, in these [dimensionless] units).

**Table 4:**
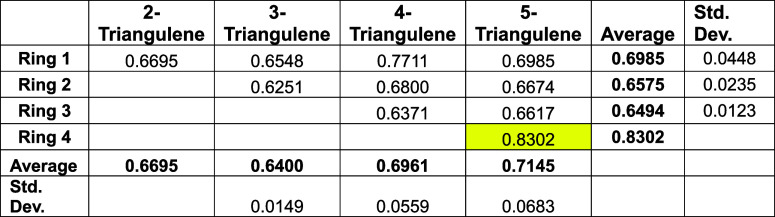
HLPM^1,2^ Topological Ring Currents (Expressed as a Ratio to the Benzene Value) in 2-Triangulene to 5-Triangulene

With the exception of the smallest member of the triangulene series (2-triangulene), there is here an identical relationship between the number of nonbonding orbitals (μ) and the number (*s*) of oxidation states having identical ring currents, as was found with the Clar’s goblet series, namely, *s* = 2 μ + 1, but, this time, with the restriction that the relationship applies only when *n* > 2.

(The data in the right-hand column of Table 5 are from calculations analogous to those in Table 2 involving multiple oxidation states, which, for reasons of brevity, are not explicitly displayed here.) A pattern emerges from Table 5; the order of the radical is equal to the number of nonbonding orbitals, that is to say,

**Table 5:** Numbers (μ) of Nonbonding Orbitals and Numbers (s) of Oxidation States Having Identical Ring Currents in the *n*-Triangulenes; *n* = 2–5

*n*-triangulene	no. (μ) of nonbonding orbitals	no. (*s*) of oxidation states having identical ring currents
2-triangulene	1	2
3-triangulene	2	5
4-triangulene	3	7
5-triangulene	4	9

2-triangulene is a monoradical and has 1 nonbonding orbital;

3-triangulene is a diradical and has 2 nonbonding orbitals;

4-triangulene is a triradical and has 3 nonbonding orbitals; and

5-triangulene is a 4-fold radical and has 4 nonbonding orbitals.

### Ring Currents in Some Truncated Triangulenes

Another series of structures that offers scope for application of the open-shell version^2^ of the HLPM (“topological”)^1,12^ approach to ring current calculations is created by taking the triangulenes and excising the bottom ring (Ring 1 in Figure 7), thereby resulting in what we call the “truncated” triangulenes, which are illustrated in Figure 8.

**Figure 8:**
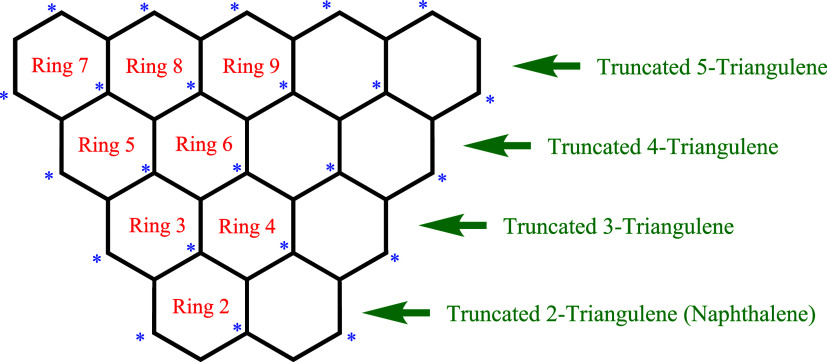
Schematic representation and ring labelings for several homologues of some truncated triangulenes (truncated 2-triangulene to truncated 5-triangulene). (The first member of the series, the two-ring closed-shell system, truncated 2-triangulene, is better known as naphthalene.).

Table 6 shows the trends in the ring currents for the various truncated triangulenes for any given ring.

**Table 6:**
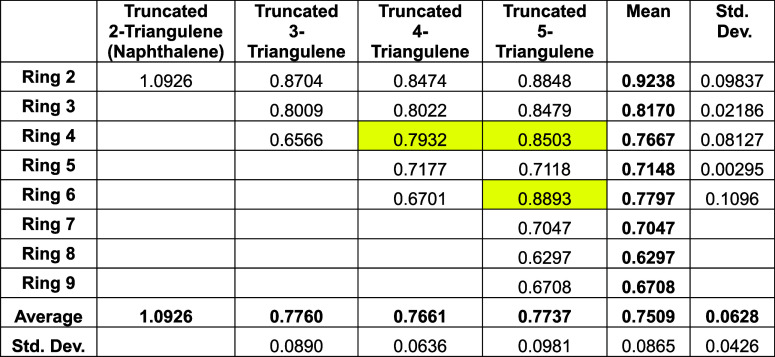
HLPM^1,2^ Topological Ring Currents in Truncated 2-Triangulene to Truncated 5-Triangulene

Again, the yellow areas indicate rings that are entirely surrounded by other rings. (As a historical footnote, it may be noted in passing that the value of 1.093 for the ring current in naphthalene, reported in the lower part of Table 5, is entirely in accordance with those reported (to the same accuracy) in the classic literature: London[Bibr ref4] (1937) and McWeeny[Bibr ref3] (1958).)

It can be seen in Figure 9 that the ipso-centric *ab initio* ^8^ and pseudo-π^7^ approaches produce similar qualitative results (compare Figure 9a,b and Figure 9c,d).

**Figure 9:**
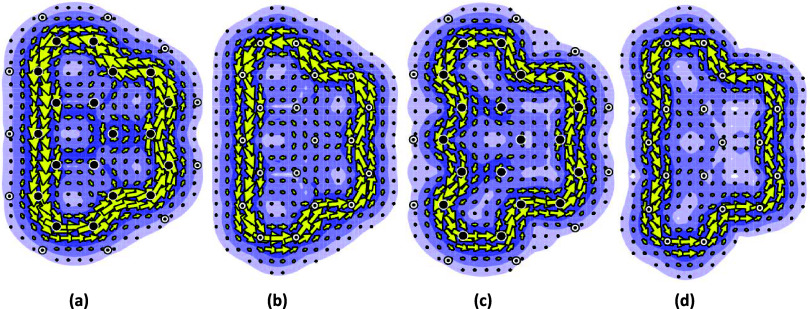
Current maps from an ipso-centric approach^8^ with π-electrons only and from the pseudo-π method^7^ for the dication and dianion of truncated 3-triangulene. (a) Ring current map for the dication derived from just the π-orbitals, using the *ab initio* method;^8^ (b) ring current map for the dication using the pseudo-π method;^7^ (c) ring current map for the dianion derived from just the π-orbitals using the *ab initio* method;^8^ and (d) ring current map for the dianion using the pseudo-π method.^7^

### Comparison of 4-Goblet with a Notional Coupling Together of Two Truncated 4-Triangulenes

We now return to a consideration of HLPM topological ring currents and observe that, as previously noted, the central rings in the goblet structures have a ring current significantly lower than those of the other rings. By comparing (in Figure 10) the currents associated with rings in the corresponding positions in *n*-goblet with those in the respective rings in the related truncated *n*-triangulene, it can be seen that the patterns of ring currents in the corresponding rings of the two conjugated systems are qualitatively similar. The sum of all of the ring currents in the nine rings on the left-hand side of 4-goblet (that is, omitting, for the moment, the central ring, with its current of 0.2088) is 6.6390, while the total ring current in all nine of the corresponding rings in truncated 4-triangulene amounts to 6.8680, a difference of some 3.4%. It may be remarked in passing that by virtue of eq 24 on p 218 of ref 36, and the fact that all ring areas are here assumed strictly to be equal to the ring area of benzene, the figure of 2 × 6.6390 + 0.2088 ∼ 13.5 represents what would be calculated by means of the original London theory^4^ to be an estimate of the total *π-electron diamagnetic susceptibility* of 4-goblet, perpendicular to its molecular plane, when that quantity is expressed as a *ratio* to the benzene diamagnetic susceptibility, calculated by the same method.^4,36^

**Figure 10:**
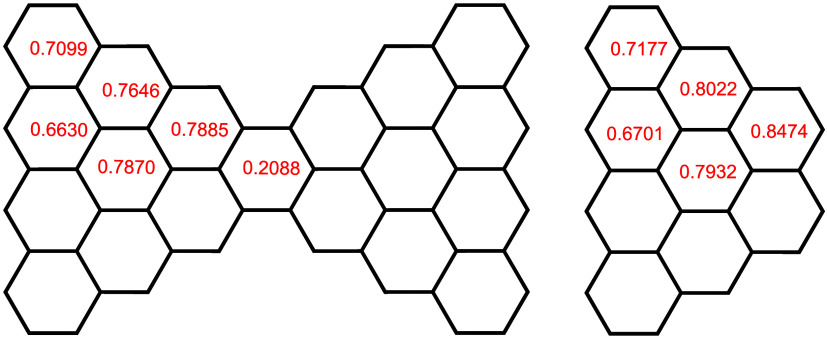
Topological ring currents in 4-goblet (left-hand side) and truncated 4-triangulene (right-hand side).

From Table 7, it may be observed that, as the sizes of triangulenes and truncated triangulenes are increased by one, the number of nonbonding orbitals increases by the same amount. However, in the goblet series, as the size of the goblet is increased by one, the number of nonbonding orbitals increases by two—2, 4, 6, ..., etc. This is apparently consistent with the notion that a Clar’s goblet may formally be considered to result from conjoining two isolated truncated triangulenes, as can be seen from Figure 10.

**Table 7:** Numbers (μ) of Nonbonding Orbitals and Numbers (*s*) of Oxidation States Having Identical Ring Currents in Truncated *n*-Triangulenes; *n* = 2–5

truncated *n*-triangulene	no. (μ) of nonbonding orbitals	no. (*s*) of oxidation states having identical ring currents
truncated 2-triangulene	0	0
truncated 3-triangulene	1	3
truncated 4-triangulene	2	5
truncated 5-triangulene	3	7

Again, from Table 7, the correlation between the number (μ) of nonbonding orbitals and the number (*s*) of oxidation states having the same ring currents is seen to be as before, namely, *s* = 2μ + 1. Table 7 shows, however, that as was the case in earlier correlations (Tables 3 and 5), this connection does not hold for the smallest of the series, truncated 2-triangulene. It should, though, be noted that, unlike the other structures listed in Table 7, truncated 2-triangulene, being, in reality, better known as naphthalene, is not an open-shell radical at all but is in fact a normal, “well-behaved”, closed-shell benzenoid hydrocarbon, whose π-electron magnetic properties were long ago studied in the classical literature by London^4^ and McWeeny.^3^

## Conclusions

The HLPM approach^1^ for calculating topological ring currents and bond currents in π-electron-conjugated systems, which was recently extended^2^ to make it applicable to open-shell structures, has been applied to two neutral classical radicals—Clar’s goblet and triangulene—as well as some of their early homologues and several of their other oxidation states. This modification was effected by applying the device^2^ of “Configuration State Averaging”, otherwise known as “Fractional Occupation” and “Electron Smearing”. The study was widened to other oxidation states of the 3-goblet, with specific emphasis on the monoanion, the dianion, the monocation, and the dication. This resulted in the remarkable finding that the topological ring currents in the above-mentioned four charged species *are all identical with the respective ring currents calculated for neutral 3-goblet*. This observation is reminiscent of a similar, but more limited, phenomenon that we observed some years ago in the context of certain neutral and dianionic *altans that are nonalternant species*.^27,28^ Analogous patterns of topological ring currents were observed among the other six oxidation states of 3-goblet (−4 → −6 and +4 → +6), as shown in Table 2.

It is important to note that Myrvold et al.^6^ emphasized that the applicability of corollaries 1–3 of theorem 1 in ref 6 has been established only for bipartite graphs/alternant hydrocarbons. As just noted, however, we have previously shown by explicit computation^27,28^ that certain so-called *altan* species have the property that the neutral structure and the dianion bear identical ring currents, despite the fact that these *altans* are nonalternant systems because they contain odd-membered rings (necessarily introduced in the course of the “altanization” process).^27,28^ This phenomenon therefore remains unexplained, and we intend to investigate it further.

The calculated topological ring currents for some of Clar’s *n*-goblets and certain *n*-triangulenes, together with several oxidation states of them, were compared with pictorial current maps obtained by means of *ab initio* ^8^ and pseudo-π^7^ formalisms; encouraging qualitative agreement was noted, overall, between the two.

Finally, the HLPM formalism^1,2^ was also applied to several homologues of a series of triangulenes with one ring (the bottom ring of the right-hand structure in Figure 1) excised; these entities are referred to as “truncated” triangulenes and it has been shown that, from the point of view of their respective constituent ring currents, Clar’s 4-goblet may be regarded as being formed when two copies of truncated 4-triangulene are notionally coupled together, thereby creating a new (central) ring, as shown in Figure 10.

This study confirms the conclusion that was drawn in ref 2 in the specific context of Clar’s goblet, namely, that the Hückel–London–Pople–McWeeny (HLPM) method^1,2,12^ can now be used to calculate topological π-electron ring currents in arbitrary conjugated hydrocarbons (whether they be open-shell or closed-shell and alternant or nonalternant) under the following conditions:

(a) on the assumption that the structure in question is geometrically planar,
(b) from a knowledge of the way in which the carbon atoms in the species are connected to each other by σ-bonds, for example, from a knowledge of the structure’s molecular graph,^1^ and
(c) from a knowledge of assumed, or actual, areas of the several individual rings of the conjugated structure under study.
